# Interdisciplinary approaches to predicting evolutionary biology

**DOI:** 10.1098/rstb.2022.0042

**Published:** 2023-05-22

**Authors:** Justin Crocker, Joshua L. Payne, Aleksandra M. Walczak, Patricia J. Wittkopp

**Affiliations:** ^1^ European Molecular Biology Laboratory, Heidelberg 69117, Germany; ^2^ Computational Biology Group, Institute of Integrative Biology, Building CHN H 75.1, Universitätstrasse 16, CH-8092 Zurich, Switzerland; ^3^ Swiss Institute of Bioinformatics, Quartier Sorge - Batiment Amphipole, 1015 Lausanne, Switzerland; ^4^ Laboratoire de Physique de l'École Normale Supérieure, CNRS, PSL University, Sorbonne Université, and Université Paris Cité, 75005 Paris, France; ^5^ Ecology and Evolutionary Biology, University of Michigan, Ann Arbor, MI 48109-1382, USA

## 1. Predicting evolution

What is predictable in evolutionary biology? Can we predict the most likely paths of evolution for molecules or gene-regulatory networks? How do population and community structures shape evolution? Can we predict the evolution of complex developmental systems or ecological communities?

Two hundred years into the exploration of evolution, the answer to these questions remains actively debated. Because evolution is shaped by multiple stochastic forces and rare events acting across molecular, population and environmental scales, there has been a skeptical view on the predictability of biological systems. Much of the skepticism has been warranted, stemming from a field that was, for a long-time, dominated by sparse empirical data or a limited number of mechanistic case studies.

Now the face of evolutionary biology is rapidly changing [1–3]. Advances in genome sequencing are providing unprecedented insights into biological mechanisms and evolutionary processes. From massively parallel evolution experiments, high-throughput sequencing and large-scale phenotypic assays, we now have an unprecedented amount of empirical evolutionary information [4]. New genome editing approaches are opening up new experimental avenues no longer limited by ‘chance’ [5], and these approaches allow us to empirically describe and quantify the types of phenotypic variation generated by genetic variation. In parallel, computational and theoretical approaches are providing new insights in our understanding of these new data and giving us tools to measure the chance events behind evolution. All of these elements are now converging into what we anticipate will become a predictive theory of evolution.

## 2. Introduction to the theme issue

In the light of new technical and theoretical advances, and to ensure that this new and growing body of evolutionary knowledge moves beyond descriptive levels towards mechanistic and causal accounts of biological processes, we hosted an EMBO workshop at the European Molecular Biology Laboratory in June 2021 called ‘Predicting evolution’. This meeting brought together a highly interdisciplinary group of scientists with the goal of integrating theoretical and experimental research into a coherent focus on ‘Predicting evolution’.

The conference explored the evolution of biological systems at different levels: from genes and molecules to organism development and ecology. As such, we invited leaders in their respective fields across various scales of evolution: molecular, network, microbial, developmental and community. The meeting explored biology at the interface of evolution, quantitative genetics, development and systems biology. In this theme issue, many of the attendees present the core ideas and main topics that emerged from discussions held at the meeting.

### (a) The predictable genome

To what extent can variation in phenotypic traits such as disease risk be accurately predicted in individuals? Accurate phenotype predictions based on genetic information is a long-standing goal across biological systems, with wide-ranging implications. However, the ability to accurately predict phenotypic variation from individual genomic sequences remains a challenge.

Epistasis, when biological components interact, is one complication in accurate phenotype prediction, as interactions between mutations add substantial complexity to adaptive landscapes. In their review, Diaz-Colunga *et al.* [6] explore the role of global epistasis in fitness landscapes. Patterns of global epistasis, in which the fitness effect of a mutation is well-predicted by the fitness of its genetic background, may aid in efforts to reconstruct fitness landscapes and infer evolutionary trajectories. To this end, the authors reconcile simple geometric reasoning with recent mathematical analyses, using these to explain why different mutations in an empirical landscape may exhibit different global epistasis patterns.

With a similar focus on epistasis, WK-G Daalman *et al.* [7] use coarse-grained molecular models to explore phenotypes predicted from budding yeast genotypes. Their research provides an example illustrating how unlikely evolutionary trajectories can become more accessible. The tractability of their biophysically inspired model suggests that such a bottom-up phenotype modelling approach is possible. With a related focus, in their research article, A-H Ghenu *et al.* [8] explore the effects of epistasis in response to environmental interactions. The authors reveal that, although epistasis may reduce the predictability of evolution in benign environments, evolution may be more predictable in adverse environments.

Adaptive processes often rely on new mutations, which can be strongly influenced by predictable biases in mutation. To explore how mutational bias shapes evolutionary biology, Cano *et al.* [9] provide an overview of existing theory and evidence for such mutation-biased adaptation. They consider the implications of these results for the problem of prediction in regard to topics such as the evolution of infectious diseases, cancer and other kinds of somatic evolution, as well as resistance to biochemical agents. In an experimental approach, Horton *et al.* [10] explore how a population will likely navigate a genotype–phenotype landscape considering selection in combination with mutation bias, which can skew the likelihood of following a particular trajectory. Using motile mutants evolved from ancestrally non-motile variants of the microbe *Pseudomonas fluorescens*, of which one trajectory exhibits significant mutation bias, the authors reveal that transient mutation bias can facilitate rapid and predictable ascension to the strongest observed phenotype. Together, these bodies of work suggest that empirical knowledge of mutational biases is likely to improve and that this knowledge is readily applicable to the challenges of short-term prediction.

### (b) Gene-regulation and networks

Heritable variation in gene expression is common within and among species and is known to contribute to phenotypic diversity [11]. Mutations affecting either *cis*- or *trans*-regulatory sequences controlling gene expression give rise to variation in gene expression, and natural selection acting on this variation causes some regulatory variants to persist in a population. Patricia Wittkopp [12] highlights the foundational work on the *TDH3* gene in *Saccharomyces cerevisiae* and how this system has revealed properties of *cis*- and *trans*-regulatory mutations including their relative frequency, effects, dominance, pleiotropy and fitness consequences. Comparing these mutational effects to the effects of polymorphisms in natural populations, they have inferred selection acting on expression level, expression noise and phenotypic plasticity.

Pleiotropy, the effect of an allele on multiple traits, is thought to enhance repeatability by constraining the number of available beneficial mutations. Ruelens *et al.* [13] address the interaction of gene pleiotropy and mutation type on evolutionary repeatability in a meta-analysis of experimental evolution studies with *Escherichia coli*. They show that non-disruptive mutations in highly pleiotropic genes yield the largest fitness benefits since they contribute more to parallel evolution, especially in large populations. These findings illustrate the importance of considering genetic architecture together with mutation type for understanding evolutionary repeatability.

Rapid enhancer and slow promoter evolution have been demonstrated through comparative genomics. However, it is not clear how this information is encoded genetically and if this can be used to place evolution in a predictive context. Li *et al.* [14] explore the evolutionary capacity of promoter variation by surveying an unbiased mutation library for three promoters in *Drosophila melanogaster*. They find that mutations in promoters have no effect on spatial patterns of gene expression, only on transcriptional levels. As such, developmental promoters may encode robust transcriptional outputs allowing evolvability through the integration of diverse developmental enhancers.

### (c) Predicting population and community evolution

Is it possible to predict the evolution of populations or communities? At an ecological level, most species belong to communities where their interactions can give rise to emergent community-level properties. Understanding and predicting how these properties change over time is a primary goal in ecology, with important implications for sustainability and human health. Venkataram & Kryazhimskiy [15] review studies of the evolution of both natural and experimental communities and make the case that community-level properties at least sometimes evolve repeatably. They argue that quantifying repeatability at the community level is not only critical to enriching our fundamental understanding of evolution and ecology but it will also help us to predict eco-evolutionary dynamics.

With a similar focus on complex coevolutionary processes, Mazzolini *et al.* [16] explore the evolution of the human immunodeficiency virus (HIV), where the virus tries to escape the continuously adapting host immune system. Using a longitudinal dataset of 10 HIV-infected people, where both the B-cell receptors and the virus are sequenced, the authors develop a simple model of antagonistically evolving populations, whereby one population has had time to sweep while the second cannot start a counter-sweep, leading to the observed anti-correlation. This study demonstrates the power of deeply sequenced communities and underscores the power of predictive models that can lead to new biological insight. In the future, similar predictive analysis can be applied to a broadening range of systems built on increasingly diverse data and methods.

How predictable is adaptation from standing genetic variation? An exciting approach to forecasting evolutionary change is the use of laboratory evolution for systems that can be run in multiple replicates and be fully sequenced. To this end, Schlötterer [17] reviews the evidence for parallel evolution in the fruit fly *Drosophila*, the best-studied obligatory outcrossing model for adaptation from standing genetic variation. He argues that selected phenotypes consistently respond in a very predictable way, but allele frequency changes are much less predictable. This implies that predicting adaptive genomic response requires a good understanding of the adaptive architecture in ancestral populations. With a focus on the impact of population size on adaptation, Servajean & Bitbol [18] find that early adaptation in rugged fitness landscapes can be more efficient and predictable for relatively small population sizes than in the large-size limit.

## 3. Concluding remarks

Across biological systems, we are in the midst of a transition from descriptive evolutionary studies to a discipline that has the data needed to make informed predictions about mechanisms of evolutionary change. In this theme issue, we focus on a range of evolutionary modes of evolution: from a fast, strong selection of microbial populations to multicellular developmental systems. As the papers in this theme issue demonstrate, the integration of both theoretical and empirical techniques from a variety of disciplines will allow us to pursue a more comprehensive theory of evolution.

Work in the coming years will explore how predictability plays out across such biological scales, including populations, communities and ecology. The approaches presented here also open up a way to set limits on predictability and see how molecular and ecological interactions and the inherent randomness of evolutionary processes come together to determine a prediction horizon. Such an integrative approach will encourage a unifying view across diverse organisms based on shared evolutionary principles. Importantly, optimizing predictions is a powerful means to learn the evolutionarily relevant functions of systems. These studies will provide insight into basic evolutionary principles that will, in turn, enhance our ability to implement rational designs for living systems, with wide-ranging implications for medicine [19] and public health [20]. By bringing together work on diverse approaches and systems, we hope this theme issue stimulates future discussion and research on the topic of predicting evolutionary biology.

## Data Availability

This article has no additional data.
